# Entrapment of the Fastest Known Carbonic Anhydrase with Biomimetic Silica and Its Application for CO_2_ Sequestration

**DOI:** 10.3390/polym13152452

**Published:** 2021-07-26

**Authors:** Chia-Jung Hsieh, Ju-Chuan Cheng, Chia-Jung Hu, Chi-Yang Yu

**Affiliations:** 1 Department of Chemical Engineering and Biotechnology, Tatung University, Taipei 104327, Taiwan; cc1235896te@gmail.com; 2 Beta Biosystem Co., Ltd., Taichung 420099, Taiwan; chuan0903@gmail.com; 3 Department of Materials Engineering, Tatung University, Taipei 104327, Taiwan; cjhu@ttu.edu.tw

**Keywords:** biomimetics, biosilicification, carbonic anhydrase, carbon sequestration, *Sulfurihydrogenibium azorense*

## Abstract

Capturing and storing CO_2_ is of prime importance. The rate of CO_2_ sequestration is often limited by the hydration of CO_2_, which can be greatly accelerated by using carbonic anhydrase (CA, EC 4.2.1.1) as a catalyst. In order to improve the stability and reusability of CA, a silica-condensing peptide (R5) was fused with the fastest known CA from *Sulfurihydrogenibium azorense* (SazCA) to form R5-SazCA; the fusion protein successfully performed in vitro silicification. The entrapment efficiency reached 100% and the silicified form (R5-SazCA-SP) showed a high activity recovery of 91%. The residual activity of R5-SazCA-SP was two-fold higher than that of the free form when stored at 25 °C for 35 days; R5-SazCA-SP still retained 86% of its activity after 10 cycles of reuse. Comparing with an uncatalyzed reaction, the time required for the onset of CaCO_3_ formation was shortened by 43% and 33% with the addition of R5-SazCA and R5-SazCA-SP, respectively. R5-SazCA-SP shows great potential as a robust and efficient biocatalyst for CO_2_ sequestration because of its high activity, high stability, and reusability.

## 1. Introduction

Industrialization in the past few centuries has increased the concentrations of greenhouse gases dramatically. CO_2_ is the most emitted greenhouse gas; the global annual fossil CO_2_ emission (including fossil fuel use, industrial processes, and product use) has increased 68% from 1990 to 2019 [1]. In order to alleviate the climate changes associated with CO_2_ emission, a number of CO_2_ mitigation methods have been developed. Conventional CO_2_ capture methods such as physical absorption and chemical absorption are quite effective, but each method has its own limitations [2]. The physical absorption method has a large absorption capacity, but it is only suitable for high CO_2_ partial pressure and the capture rate is low. The chemical absorption is the most widely used CO_2_ capture method, but it requires a large amount of energy and thus the overall cost is high.

Biomimetic carbon sequestration using carbonic anhydrase (CA, EC 4.2.1.1) has drawn a lot of attention as an alternative CO_2_ mitigation method because the process is both green and economical [3]. CA catalyzes the hydration of CO_2_, in which CO_2_ is converted to bicarbonate with the concomitant release of a proton [4]; the hydration is considered one of the rate limiting steps in CO_2_ sequestration in aqueous solutions [5]. By reacting with a calcium ion, the bicarbonate is then converted to calcium carbonate, which can be easily collected and stored or used in industry.

CA is a zinc metalloenzyme which can be found in almost all living organisms [6]. The biomimetic carbon sequestration is often performed at an elevated temperature (for instance, capturing CO_2_ in flue gas) and the subsequent mineralization process is carried out at alkaline pH; therefore, thermo-alkali-stable CAs are preferable. Two highly thermo-alkali-stable CAs isolated from thermophilic archaea *Sulfurihydrogenibium yellowstonense* YO3AOP1 (SspCA) and *S. azorense* (SazCA) have drawn much research interest recently because of their unique biochemical properties. SspCA possesses exceptional thermal stability; the enzyme retains most of its activity even after 3 h incubation at 100 °C [7]. SazCA is the fastest enzyme known for the CO_2_ hydration reaction with a k_cat_ of 4.4 × 10^6^ s^−1^; however, it is less thermally stable than the SspCA [8]. In addition, both CAs are alkali-stable and not inhibited by common anions present in flue gas such as NO_2_^−^, NO_3_^−^, and SO_4_^2^^−^ [8,9]. These unique properties make both CAs ideal catalysts for the biomimetic carbon sequestration. However, like many enzymatic processes, the stability and reusability of these fragile biocatalysts limit their industrial applications. Immobilization is a useful technique for improving enzymes’ industrial applicability; various supports have been reported for immobilizing CA, including mesoporous silica, chitosan-alginate beads, electrospun nanofibers, and PVDF membranes [10]. Most of the immobilization supports require pre-activation steps except for those immobilizing the biocatalysts via physical adsorption; however, the weak binding force of adsorption often leads to severe enzyme leaching. A rapid and robust method for immobilizing these thermo-alkali-stable CAs is in need.

Biosilicification, a common mineralization phenomenon in living organisms, is often observed in single-celled organisms such as diatoms. Silaffins, highly post-translationally modified proteins isolated from the diatom *Cylindrotheca fusiformis*, are crucial to the formation of its silica cell wall [11]. The R5 peptide (H_2_N-SSKKSGSYSGSKGSKRRIL-COOH), one of the repeating sequences observed in the *C. fusiformis* silaffin-1A, has been shown to catalyze the formation of silica nanoparticles at ambient temperature and neutral pH with no post-translational modifications [12]. The biomimetic silica formed by the R5 peptide has been applied to enzyme immobilization [13,14,15]; the process is facile and rapid, and the mild reaction conditions allow the enzyme molecules retain most of their activities. Although the R5 peptide is widely applied in biotechnology, the role of its structure in the silica formation process is still unclear. Lutz et al. showed that R5 has some secondary structure (mainly β-sheet) at interfaces and maintains a defined conformation within silica sheets [16]; however, others reported that R5 has no ordered secondary structure in solution [17].

In this work, we improved the reusability and stability of SazCA, the fastest known CA, by entrapment in biomimetic silica. A fusion enzyme composed of the R5 peptide and SazCA, namely R5-SazCA, was constructed. R5-SazCA was further entrapped in silica to improve its biochemical characteristics via the silica-forming ability of the R5 peptide. To the best of our knowledge, such a fusion enzyme has yet to be reported. The biochemical characteristics of the free and entrapped enzymes were determined and compared; in addition, the feasibility of using R5-SazCA for CO_2_ sequestration was also demonstrated.

## 2. Materials and Methods

### 2.1. Bacterial Strains and Vector Construction

**Figure 1:**
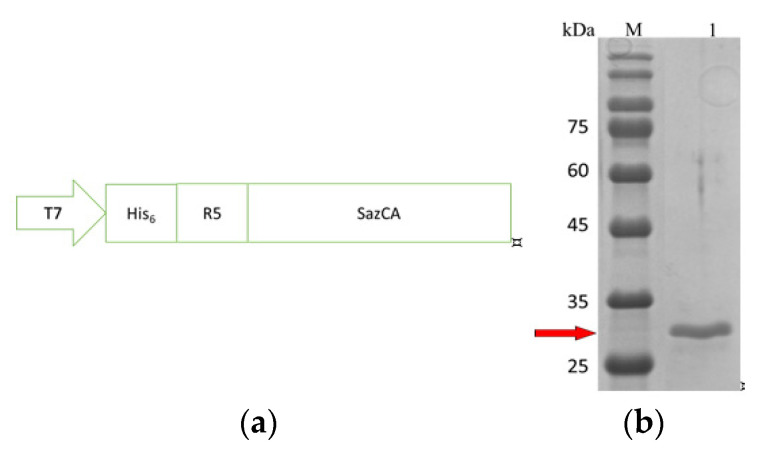
Gene construction and purification of R5-SazCA. (**a**) Schematic of the gene; (**b**) SDS-PAGE; lanes: M, marker; 1, purified R5-SazCA.

All DNA work was performed using *E. coli* DH5α (YB Biotech, Taipei, Taiwan) based on standard protocols. Fusion enzyme expression was conducted in *E. coli* BL21 (DE3) also purchased from YB Biotech. The SazCA gene was synthesized by Protech Technology Enterprise (Taipei, Taiwan) and inserted into a pET-28a(+) vector via the restriction sites of *Nde*I and *Xho*I (pET-28a(+)-SazCA). The DNA sequence encoding the R5 peptide was inserted by Gibson assembly. The PCR product containing the SazCA gene was amplified by the primers: 5′-ATCCTACTCGGGATCCAAGGGTTCCaagcgtcgcatcttgatggcggaagtgcaccactg-3′ (forward) and 5′-AGCCGGATCTCACTCGAGGTTGCTttccagaat-3′ (reverse); sequences denoting the R5 peptide and the overlapping region are underlined and capitalized, respectively. The PCR product containing the vector was amplified by the primers: 5′-AGCAACCTCGAGTGAGATCCGGCTgctaacaaa–3′ (forward) and 5′-GGAACCCTTGGATCCCGAGTAGGAtccggatttcttggaggaatggctgccgcgcggcac-3′ (reverse); the denotations are the same as previously described. One microliter of the PCR product containing the SazCA gene was mixed with 9 μL of the PCR product containing the vector and 10 μL of 2 × Gibson mix (NEB, Ipswich, MA, USA), the mixture was incubated at 50 °C in a thermocycler for 1 h and the reaction resulted in pET-28a(+)-R5-SazCA. The R5-SazCA gene has a hexahistidine (His_6_)-tag sequence at its N-terminus as indicated in Figure 1a.

### 2.2. Expression and Purification of R5-SazCA

The *E. coli* BL21(DE3) transformants carrying the pET-28a(+)-R5-SazCA plasmids were cultured in a 300 mL flask containing 100 mL of LB medium supplemented with 30 μg/mL kanamycin at 37 °C with rotary shaking at 180 rpm. Protein expression was induced with 1 mM IPTG when OD_600_ reached 0.6–0.8; 0.5 mM ZnSO_4_ was also supplemented as a source of Zn^2+^, which is required for the enzyme activity. The cells were further cultured for 6–8 h at 25 °C, and then the cells were collected by centrifugation at 8000× *g* for 30 min at 4 °C. The cell pellet was lysed with the lysis buffer (20 mM Tris-sulfate containing 300 mM NaCl and 10 mM imidazole, pH = 8.3) supplemented with EDTA-free protease inhibitor (SIGMA, St. Louis, USA) The cells were also mixed with approximately one-third suspension volume of 0.1 mm glass beads (BioSpec Products, Bartlesville, USA); the mixture was vortexed for 1 min and then rested on ice for 5 min. The cycle was repeated 3–5 times until the supernatant turned slightly clear. The cell debris was removed by centrifugation at 8000× *g* at 4 °C for 20 min, and then the lysate was subject to purification using Ni-NTA His-Bind Resin Chromatography kit (Merck, Darmstadt, Germany) according to the supplier’s protocol at 4 °C. The purified R5-SazCA was then desalted against the storage buffer (20 mM Tris-sulfate, pH = 8.3) using a PD-10 column. The enzyme concentration was determined using Bradford assay with BSA as standards.

### 2.3. Hydratase Activity Assay

The assay was based on the method reported by others with modifications [7]; an indicator, bromothymol blue, was used to monitor the pH change resulting from the proton released during the conversion of CO_2_ to bicarbonate catalyzed by CA. The CO_2_-saturated water was prepared by bubbling CO_2_ into 100 mL deionized water chilled in an ice-water bath for roughly 3 h. Ten to fifty microliters of the enzyme solution were added to a test tube containing 1 mL of 25 mM Tris-HCl, pH = 8.3, with bromothymol blue already chilled in an ice-water bath. The reaction was initiated by adding 1 mL of the CO_2_-saturated water to the test tube and immediately a stopwatch was started. Using 25 mM Tris-HCl, pH = 6.3, with bromothymol blue as a color reference, the time required for the mixture to change from blue (pH = 8.3) to yellow (pH = 6.3) was recorded. The time required for the color change of an uncatalyzed reaction was also recorded by replacing the enzyme solution with an equivalent volume of storage buffer. One Wilbur–Anderson unit (WAU) is defined as (T_0_ − T)/T, where T_0_ and T are the time required for the pH decreasing from 8.3 to 6.3 for the uncatalyzed and catalyzed reaction, respectively.

### 2.4. Esterase Activity Assay

The esterase activity was determined at room temperature using *p*-nitrophenylacetate (*p*-NpA) as the substrate [7]. Ten microliters of enzyme solution were added to a mixture containing 0.3 mL of freshly prepared 3 mM *p*-NpA and 0.7 mL of 15 mM Tris sulfate, pH = 7.6, and then A_348_ was monitored for 5 min with a Jasco V-550 spectrophotometer. One unit is defined as an increase of 0.03 at A_348_ in 5 min; the increase in A_348_ was corrected against an uncatalyzed reaction.

### 2.5. Entrapment of R5-SazCA in Silica Nanoparticles

In order to form the silica nanoparticles, 152 μL of tetramethyl orthosilicate was first acid-hydrolyzed to silicic acid by mixing with 848 μL of 1 mM HCl at room temperature for 15 min; the silicic acid needs to be freshly prepared. Silica nanoparticles were formed by mixing 900 μL of 0.5–0.7 mg/mL enzyme solution in storage buffer with 100 μL of fresh silicic acid; the pH of the resulting mixture was about 7.8. The nanoparticles containing R5-SazCA (R5-SazCA-SP) were collected by centrifugation at 10,000× *g* for 5 min followed by washing with 1 mL of storage buffer twice. The washed R5-SazCA-SP was suspended in 1 mL of storage buffer and stored at 4 °C for later use.

### 2.6. CO_2_ Sequestration

The CO_2_ sequestration was based on the process reported by Jo et al. [18]. Fifty microliters of R5-SazCA solution or R5-SazCA-SP suspension (the concentration was 0.6 mg/mL for both forms of enzymes) were mixed with 450 µL of 1 M Tris containing 20 mM CaCl_2_, pH = 11, in a cuvette; CaCO_3_ precipitation was initiated by the addition of 500 µL of CO_2_-saturated water prepared at 30 °C. The reaction was monitored by measuring A_600_ using a thermostated spectrophotometer (JASCO V-550) at 30 °C. An uncatalyzed reaction was also performed by replacing the enzyme solution with storage buffer. In order to obtain sufficient amount of CaCO_3_ for characterization, the reaction was scaled up to a total volume of 40 mL; after 5 min of incubation, the precipitates were filtered through a 0.45 μm membrane filter, and then subjected to XRD and SEM analysis after overnight drying at 70 °C in an oven. The SEM image was obtained with a Hitachi SU8000 scanning electron microscope. The XRD patterns were determined with a Bruker D2 PHASER diffractometer.

## 3. Results and Discussion

### 3.1. Expression and Purification of R5-SazCA

The His_6_-tag and the R5 peptide were fused to the N-terminus of SazCA, as shown in Figure 1a. The expression level after the IPTG induction was analyzed hourly with SDS-PAGE (data not shown); the expression level increased rapidly in the first 3 h and peaked around 7–8 h. An incubation period of 8 h after induction was selected for the production of R5-SazCA. The SDS-PAGE image of the purified R5-SazCA is shown in Figure 1b; the purified protein had almost no contaminations and the purity reached 95% as estimated using the IMAGE J software. The fusion enzyme has a theoretical molecular weight of 30,726 Da and a theoretical isoelectric point (PI) of 9.14 as calculated using online tools [19]. R5-SazCA had an averaged specific activity of 18,508 ± 710 WAU/mg (N = 4), which was very similar to that of a SazCA without the R5 peptide (also constructed, expressed and purified in our laboratory), indicating that the presence of R5 does not affect the activity. Our observation is also supported by the fact that the N-terminus of SazCA forms a random coil which is far away from its active site [20]. The determination of enzyme kinetic constants failed after several attempts; the extremely high activity of SazCA may require a stopped-flow apparatus to accurately determine these constants [8].

### 3.2. Encasuplation of R5-SazCA

**Figure 2:**
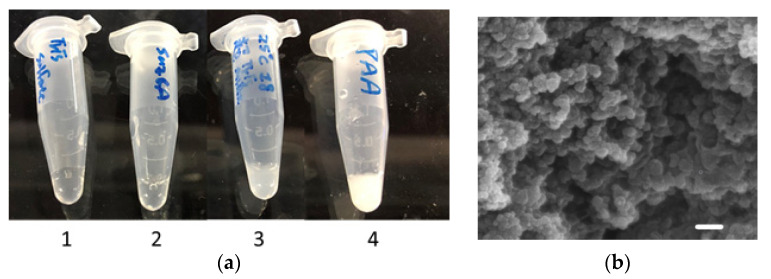
Silica nanoparticles and their morphology. (**a**) The silica-forming ability of the fused R5 peptide. One hundred microliters of freshly prepared silicic acid were mixed with 900 μL of (**1**) storage buffer; (**2**) 0.5 mg/mL SazCA in storage buffer; (**3**) 0.5 mg/mL R5-SazCA in storage buffer; and (**4**) 0.2 mM polyallylamine in storage buffer. Polyallylamine, a common catalyst for biomimetic silica formation, was used as a positive control. (**b**) SEM image of R5-SazCA-SP. The scale bar corresponds to a length of 1 μm.

The silica-forming ability of the fused R5 peptide was examined by adding freshly prepared silicic acid to the R5-SazCA solution; the silica particles formed within seconds (image 3, Figure 2a). The negative controls containing storage buffer or SazCA solution did not result in silica formation (image 1 and 2, Figure 2a), clearly indicating that the formation of silica particles was catalyzed by the fused R5 peptide. The supernatant after entrapment of R5-SazCA showed no CO_2_ hydratase activity, suggesting that most of the enzyme molecules were immobilized, and thus the entrapment efficiency (amount of entrapped R5-SazCA/amount of added R5-SazCA) was close to 100%. The high entrapment efficiency associated with the R5 peptide was also reported by others [14,18]. The specific activity of R5-SazCA-SP was 16,887 ± 411 WAU/mg (N = 4), and the activity recovery (specific activity of R5-SazCA-SP/specific activity of R5-SazCA) was 91%. The enzyme leaching from R5-SazCA-SP was examined by monitoring the hydratase activity in storage buffer up to 48 h, and no activity was observed. The silica matrix tightly entrapped the enzymes; a similar observation was made for *Neiserria gonorrhoeae* CA entrapped within biomimetic silica [18]. The morphology of R5-SazCA-SP was examined by SEM; a matrix of fused silica particles was observed (Figure 2b). Some spherical structure could be seen in part of the matrix; the diameter ranged from 320 to 580 nm with an average of 460 ± 80 nm (N = 10).

### 3.3. Effect of Temperature on Activity and Stability

The esterase activity assay was selected to study the effects of temperature on activity; the CO_2_ hydratase activity assay was not used because the CO_2_ solubility decreases with increasing temperature. Temperature affected the activities of both forms of R5-SazCA similarly (Figure S1); the activities increased as temperature increased from 50 to 80 °C. The optimal activities of 2567 ± 144 and 3213 ± 140 U/mg were both found at 80 °C for R5-SazCA and R5-SazCA-SP, respectively; the activity of R5-SazCA was enhanced by 25% after coating with biomimetic silica. However, the activities started to decline for both when the temperature was further elevated to 90 °C. The activity-temperature profile of R5-SazCA is similar to that of SazCA [8], which also has an optimum temperature of 80 °C.

**Figure 3:**
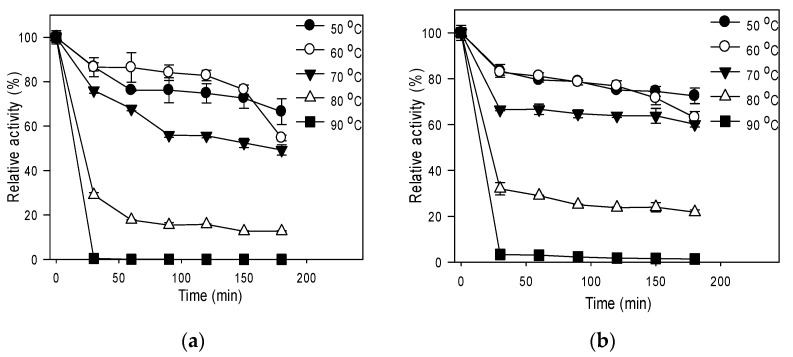
Effect of temperature on stability. (**a**) R5-SazCA; (**b**) R5-SazCA-SP. The activities before incubation were set as 100%. Aliquot of enzyme solution was assayed for its residual CO_2_ hydratase activity every 30 min.

The effect of temperature on stability is shown in Figure 3; the residual activities of R5-SazCA were 66%, 54%, and 49% after 3 h incubation at 50, 60, and 70 °C, respectively (Figure 3a). The residual activities of R5-SazCA-SP were 73%, 63%, and 60% after 3 h incubation at 50, 60, and 70 °C (Figure 3b), respectively, clearly indicating that the thermal stability was improved after immobilizing the free enzyme with biomimetic silica. At 80 and 90 °C, both forms of R5-SazCA lost most of the activities within the first 30 min; however, the immobilized form still had higher residual activities. Improved thermal stability upon entrapment in biomimetic silica has been reported for other CA [18,21,22]; the enhanced thermal stability is often attributed to the steric constraints imposed by the crosslinking-network of silica, which aid in maintaining the stereo structure of the enzyme.

### 3.4. Effect of pH on Stability

**Figure 4:**
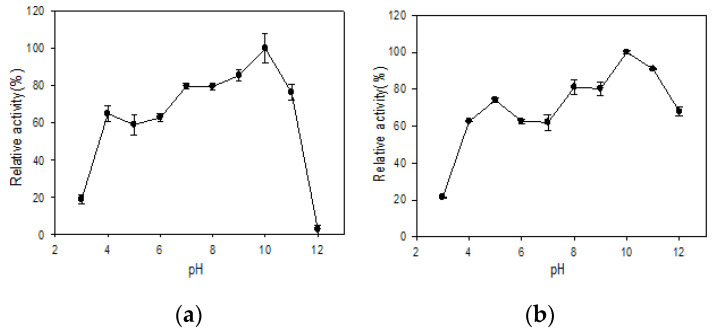
Effect of pH on stability. (**a**) R5-SazCA; (**b**) R5-SazCA-SP. The highest activity was set as 100%. The pH stability was determined by pre-incubating the enzyme solution with a buffer system at a volumetric ratio of 1:9 (enzyme: buffer system) for 30 min; the buffer system contained 150 mM glycine, 150 mM H_3_PO_4_, and 150 mM Tris-base with pH adjusted to 3–12. After incubation, the enzyme solution was assayed for its residual CO_2_ hydratase activity.

The effect of pH on stability is shown in Figure 4. Both R5-SazCA and R5-SazCA-SP were most stable at pH 10; they retained at least 59% of their activities in the range from 4 to 11. The entrapment in biomimetic silica improved the stability at pH = 11 and 12; 67% of the activity was retained for the immobilized form while the free form was almost completely inactivated at pH = 12. The improved pH stability was also reported for CA originating from cyanobacterium *Synechocystis* sp. entrapped in biomimetic silica [22].

### 3.5. Cation and Salinity Tolerance

**Figure 5:**
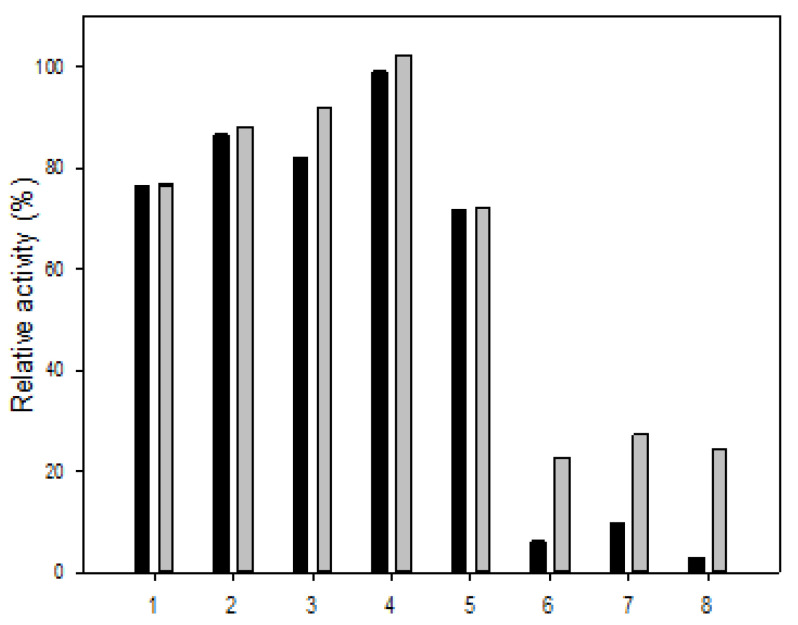
Cation tolerance of R5-SazCA and R5-SazCA-SP. 1: NH_4_^+^; 2: K^+^; 3: Na^+^; 4: Mg^2+^; 5: Mn^2+^; 6: Zn^2+^; 7: Cu^2+^; and 8: Fe^2+^. Black bar: R5-SazCA; gray bar: R5-SazCA-SP. The activities before incubation with cations were set as 100%. The enzyme solution or suspension was incubated with the cation at a final concentration of 5 mM for 30 min followed by measuring its CO_2_ hydratase activity.

The cation tolerance is shown in Figure 5. In the presence of Mg^2+^, the activities of R5-SazCA and R5-SazCA-SP were unaffected. As for NH_4_^+^, Na^+^, K^+^, and Mn^2+^, decreased activities were observed for both forms of enzymes, but at least 70% of the initial activities were retained. However, Fe^2+^, Cu^2+^, and Zn^2+^ all had severe inhibitory effects on R5-SazCA; only 6%, 9%, and 2% of the initial activities were retained, respectively. The activities under the influence of Fe^2+^, Cu^2+^, and Zn^2+^ were increased significantly to 22%, 27%, and 24% after entrapment, respectively. It is clear that the silica coating protects the enzyme from the inhibitory effects exerted by these metal ions. The inhibition caused by Cu^2+^ and Zn^2+^ has also been reported for SspCA, and the inhibition by Cu^2+^ is likely due to the possible binding of Cu^2+^ to the imidazole side chain of His residues in the active center [20,23]. The inhibition by Zn^2+^ is unexpected because it is a cofactor for SazCA; however, Zn^2+^ is inhibitory to CA from ducks at concentrations higher than 7.5 μM [24].

For industrial-scale carbon sequestration utilizing CA, a large amount of water as CO_2_ carrier is often necessary. Using seawater is more sensible in such a process as fresh water is becoming a limited resource; therefore, the salinity tolerance was examined (Figure S2). R5-SazCA and R5-SazCA-SP still retained 96% and 94%, respectively, of the initial activities even after 30 min of incubation with 6% (*w*/*v*) NaCl. Both forms of R5-SazCA demonstrated good salinity tolerance, clearly showing that they are suitable for carbon sequestration applications using seawater because its salinity is about 3.5%. SspCA also showed little loss in activity up to 6% NaCl [23], which is very similar to our results.

### 3.6. Storage Stability and Reusability

**Figure 6:**
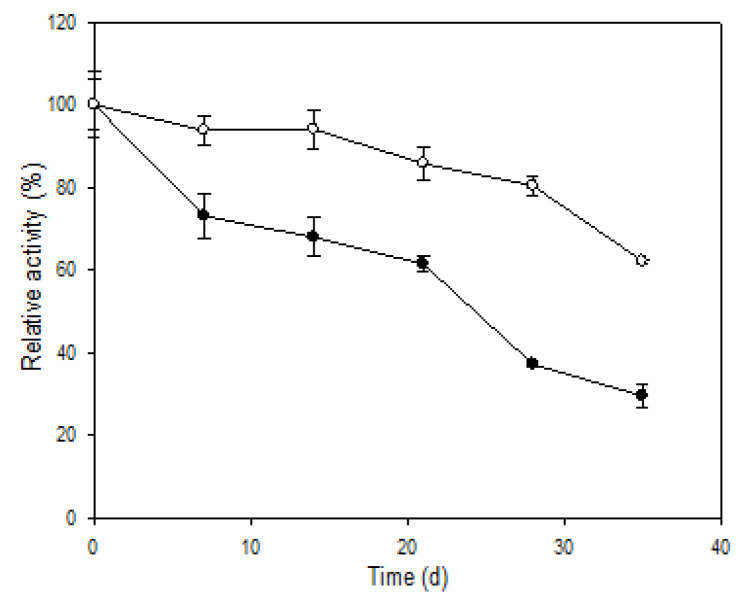
Storage stability at 25 °C. Initial activities were defined as 100%. Solid circle: R5-SazCA; open circle: R5-SazCA-SP. The residual activity was monitored weekly using the CO_2_ hydratase activity assay.

When stored at 4 °C (data not shown), two forms of R5-SazCA had similar stabilities, the residual activities remained above 90% for both after 35 days. However, the activity of R5-SazCA decreased significantly after 35 days of storage at 25 °C; only 30% of the initial activity remained (Figure 6). R5-SazCA-SP showed a much slower decrease in activity at 25 °C with 62% of enzyme activity remaining after 35 days, which was 2-fold higher than that of the free form. The results clearly demonstrate that the entrapment in silica effectively improves the storage stability of R5-SazCA.

**Figure 7:**
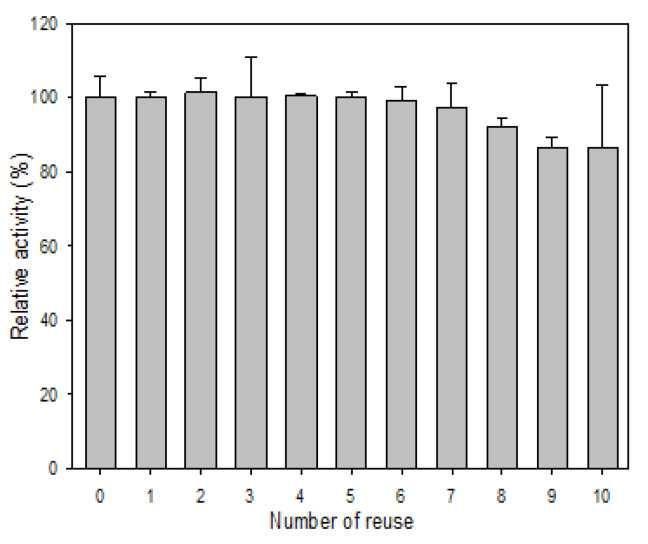
Reusability of R5-SazCA-SP. The initial CO_2_ hydratase activity was set as 100%. After each reaction cycle, R5-SazCA-SP was collected by centrifugation at 10,000× *g* at 4 °C for 5 min, followed by washing with 2 mL of storage buffer twice.

As for the reusability, R5-SazCA-SP showed almost no loss in activity during the first seven cycles of reuse (Figure 7); 86% of the activity remained even after ten cycles of reuse. The decrease in activity during recycling is partly due to the loss of silica particles in the recovery and washing process because the immobilized enzyme is very stable at 4 °C as indicated in the storage stability study. The reusability of R5-SazCA-SP is similar to that of BMC-SazCA (a fusion protein composed of an ectodomain of the human receptor-type tyrosine-protein phosphatase C, a cellulose-binding module, and SazCA) immobilized on microcrystalline cellulose beads, which showed a residual activity of 90% after ten cycles of reuse [25].

### 3.7. CO_2_ Sequestration in CaCO_3_

**Figure 8:**
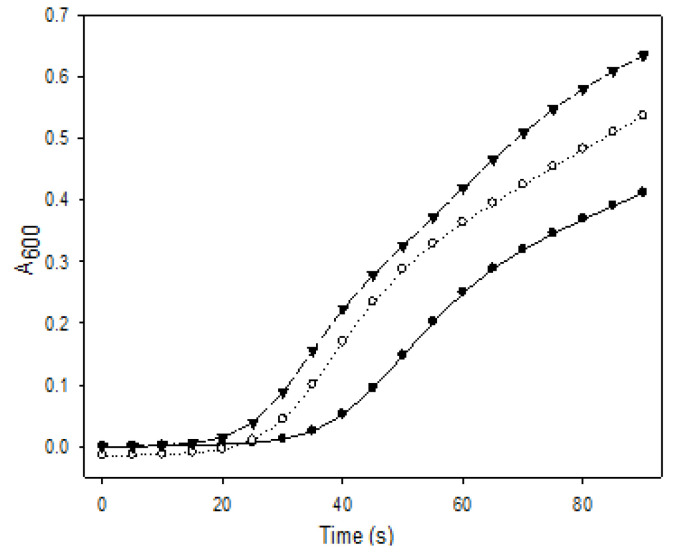
CO_2_ sequestration in CaCO_3_. Solid circle: negative control without the addition of enzyme; open circle: reaction catalyzed by R5-SazCA-SP; solid triangle: reaction catalyzed by R5-SazCA. The formation of CaCO_3_ was monitored turbidometrically at A_600_.

**Figure 9:**
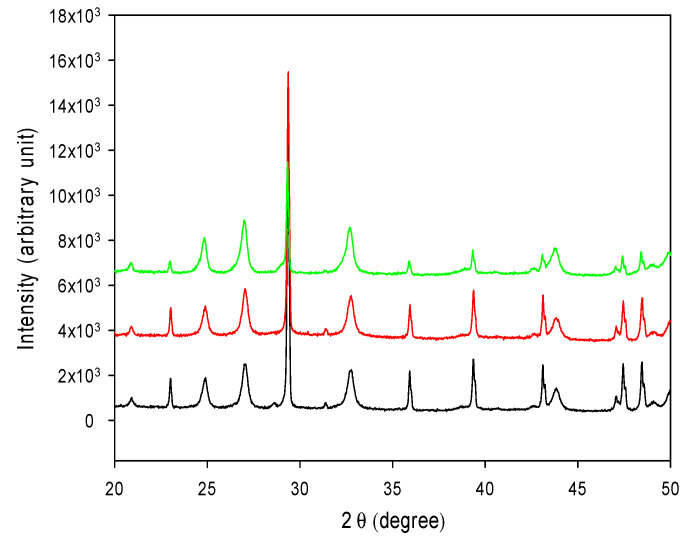
XRD patterns of the precipitated CaCO_3_. C: calcite; V: vaterite. Black: reaction catalyzed by R5-SazCA; red: reaction catalyzed by R5-SazCA-SP; green: negative control without the addition of enzyme.

**Figure 10:**
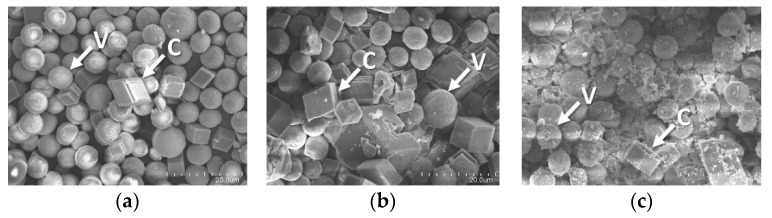
SEM images of the precipitated CaCO_3_. (**a**) Reaction catalyzed by R5-SazCA; (**b**) reaction catalyzed by R5-SazCA-SP; (**c**) negative control without the addition of enzyme. C: rhombohedral calcite; V: spherical vaterite.

The ability of converting CO_2_ to CaCO_3_ was examined for both forms of R5-SazCA; the formation of CaCO_3_ was monitored turbidometrically and the results were shown in Figure 8. The CaCO_3_ formation rate derived from Figure 8 was significantly increased by the addition of enzyme. The onset time required for CaCO_3_ formation (defined as ΔA_600_ per second > 0.001) were 16.3 s and 19.1 s when the reactions were catalyzed by R5-SazCA and R5-SazCA-SP, respectively. The onset time was shortened by 43% and 33% with the addition of R5-SazCA and R5-SazCA-SP, respectively, when compared with an onset time of 28.7 s of the negative control. The XRD patterns revealed that the precipitated CaCO_3_ were comprised of rhombohedral calcite and spherical vaterite (Figure 9), which can also be clearly seen in the SEM images (Figure 10). The crystal structure of CaCO_3_ is not altered by the addition of enzyme, as evidenced by the XRD patterns and SEM images.

## 4. Conclusions

We report a rapid and facile approach for immobilizing SazCA, the fastest CA known, and the immobilized enzyme successfully catalyzed carbon sequestration. The entrapment in biomimetic silica catalyzed by R5 has high efficiency and minimal enzyme leakage; in addition, the process can be performed under ambient conditions with no prior support-activation required. The silica matrix significantly improves the thermal and alkaline stability of SazCA, which are crucial to industrial carbon sequestration. The salinity tolerance of both forms of enzymes is adequate for applications utilizing seawater as a CO_2_ carrier. The entrapped form, R5-SazCA-SP, can be easily reused and stored at room temperature with little loss in activity up to five weeks. R5-SazCA-SP successfully accelerated the conversion of CO_2_ to CaCO_3_ without altering its crystal structure. Because of the high activity, high stability, and reusability of R5-SazCA-SP, it can serve as a robust biocatalyst for carbon sequestration. In addition, it can be applied to applications such as biosensors, synthesis of chemicals, and pollutant removal by coupling with other enzymes.

## Data Availability

Not applicable.

## Supplementary Materials

The following are available online at https://www.mdpi.com/article/10.3390/polym13152452/s1, Figure S1: Effect of temperature on activity, Figure S2: Salinity tolerance of R5-SazCA and R5-SazCA-SP.
