# Supplementary material for: Entrapment of the Fastest Known Carbonic Anhydrase with Biomimetic Silica and Its Application for CO2 Sequestration
Source: Polymers (Basel). 2021 Jul 26;13(15):2452. doi: 10.3390/polym13152452 (PMC8347136; doi:10.3390/polym13152452)
Supplement: Supplementary file 1 [file polymers-13-02452-s001.zip › polymers-1308722-supplementary.pdf]

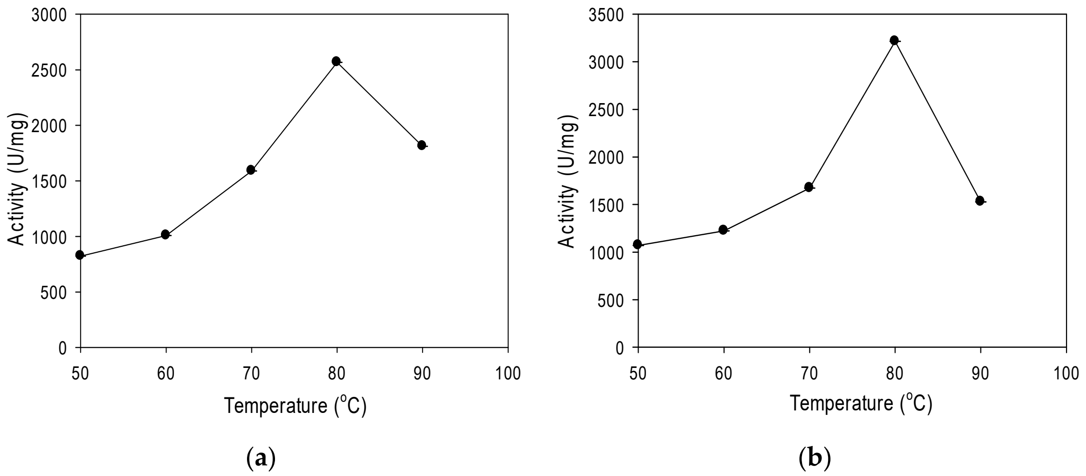

Figure S1. Effect of temperature on activity. (**a**) R5-SazCA; (**b**) R5-SazCA-SP.

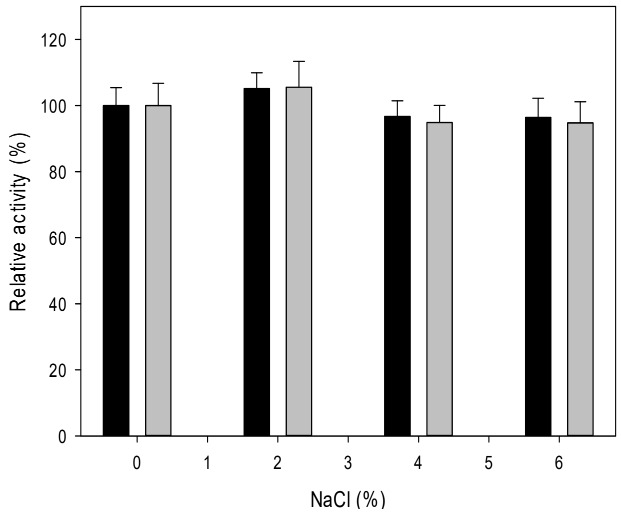

Figure S2. Salinity tolerance of R5-SazCA and R5-SazCA-SP. Black bar: R5-SazCA; grey bar: R5-SazCA-SP. The activities in the absence of NaCl were set as 100%. The salinity tolerance was examined by incubating the enzyme with 2%, 4% and 6% (w/v) of NaCl supplemented in storage buffer for 30 min followed by assaying the CO_2 hydratase activity.
